# Effects of open-label placebos in clinical trials: a systematic review and meta-analysis

**DOI:** 10.1038/s41598-021-83148-6

**Published:** 2021-02-16

**Authors:** Melina von Wernsdorff, Martin Loef, Brunna Tuschen-Caffier, Stefan Schmidt

**Affiliations:** 1 Department of Psychosomatic Medicine and Psychotherapy, Medical Center Freiburg, Faculty of Medicine, University of Freiburg, Hauptstr. 8, 79104 Freiburg, Germany; 2 Department of Psychology, Clinical Psychology and Psychotherapy, University of Freiburg, Freiburg, Germany; 3 CHS-Institut, Berlin, Germany; 4 Institute for Frontier Areas and Mental Health, Freiburg, Germany

**Keywords:** Psychology, Medical research, Clinical trials, Placebo effect

## Abstract

Open-label placebos (OLPs) are placebos without deception in the sense that patients know that they are receiving a placebo. The objective of our study is to systematically review and analyze the effect of OLPs in comparison to no treatment in clinical trials. A systematic literature search was carried out in February 2020. Randomized controlled trials of any medical condition or mental disorder comparing OLPs to no treatment were included. Data extraction and risk of bias rating were independently assessed. 1246 records were screened and thirteen studies were included into the systematic review. Eleven trials were eligible for meta-analysis. These trials assessed effects of OLPs on back pain, cancer-related fatigue, attention deficit hyperactivity disorder, allergic rhinitis, major depression, irritable bowel syndrome and menopausal hot flushes. Risk of bias was moderate among all studies. We found a significant overall effect (standardized mean difference = 0.72, 95% Cl 0.39–1.05, *p* < 0.0001, *I*^2^ = 76%) of OLP. Thus, OLPs appear to be a promising treatment in different conditions but the respective research is in its infancy. More research is needed, especially with respect to different medical and mental disorders and instructions accompanying the OLP administration as well as the role of expectations and mindsets.

## Introduction

Placebos have been the subject of many studies in the last two decades^1^ and the number of clinical trials to examine a placebo treatment as the primary intervention is rapidly growing^2^. Research has shown that symptoms can be reduced in a significant way by receiving an inert medication^3–5^. Placebos are also increasingly used in medical practice outside of clinical trials^2,6,7^. A survey in the UK revealed that 77% of general practitioners use placebos regularly^8^. Considering not only the benefits for patients (i.e. no pharmacological side effects) but also economic effects like low priced pills^9^, deceptive placebos appear to be a promising alternative to active substances in medicine.

However, the use of placebos in primary treatment raises ethical concerns because the physicians’ prescriptions may be considered to be deceptive^10^. Patients need to be informed completely, accurately and comprehensively about their treatment^11^, otherwise the essential base for a healthy relationship between physician and patient is jeopardized^12,13^. Despite some these ethical concerns, a few researchers contend that deceptive placebos are acceptable in a limited number of circumstances (e.g.^14–16^) since the therapeutic encounter can still be beneficial to the patient. Others say that physicians are still lying to patients “in order to bring about positive expectations surrounding treatment outcomes”^17^ ^p.^ ^2^ which might harm the fiduciary patient-doctor relationship. This dilemma raises the question of whether the deception in placebo treatments is coercively necessary for achieving a placebo effect.

In 1965, Park and Covi^18^ were the first researchers who examined if full transparency regarding the placebo treatment would still result in an observable placebo effect. Surprisingly, they found a reduction in symptoms even if patients knew that they received a placebo treatment with inert sugar pills. This line of research was not pursued further until the first randomized controlled trial (RCT) was published in 2008, which examined the placebo-effect without deception (open-label placebo, OLP) as a “dose-extender” in children with attention deficit hyperactivity disorder (ADHD)^19,20^. In 2010, a ground-breaking study was published by Kaptchuk et al.^21^, in which they found significant effects of OLPs in patients with irritable bowel syndrome.

Several recent reviews^22–25^ provide an overview of current advances in clinical OLP research and formulate first hypotheses as to why placebos without deception may still have beneficial effects. The general problem in the research of placebo treatments is to differentiate adequately between a *placebo effect* (effect due to the placebo treatment) and a *placebo response*. The latter refers to *all* effects found in the placebo arm of a RCT. This includes, alongside the placebo effect, also the natural tendency of the condition to improve, the statistical artifact of regression to the mean, and the Hawthorne effect due to mere attention and measurement^1^. Blease et al.^22^ discuss three methodological challenges to clinical OLP research. These are the choice of control group, potential bias due to unblinded clinican experimenters, and finally the role of the instruction accompanying the OLP administration. Control group OLPs are usually either compared to a ‘treatment as usual’ (TAU) arm or to a ‘no treatment’ (NT) arm, e.g. a wait-list condition. There is often a criticism that patients in the TAU group are not adequately monitored and that, as a result of this, the structural equivalency cannot be guaranteed^22,26^. On the other hand, wait-list controls are associated with nocebo effects in psychotherapy research^27^. But even with perfectly paralleled groups, whether TAU or NT, a significant problem remains that participants in the OLP arm and in the control group are treated differently. In one group they receive a placebo often accompanied with a positive instruction (“this placebo pill might help”) and in the control condition they don’t. This difference may result in a Hawthorne effect in the treatment arm and/or cause disappointment in the control group. Another methodological challenge is blinding. If the OLP is administered by a clinician to the patients neither of them are blinded. Thus, an OLP, defined as the administration of an inert pill with an instruction informing the patient about its inertness, cannot be seamlessly integrated into the methodology of a pharmacological RCT. Due to the information given as part of the treatment, an OLP shares aspects of psychological treatments that are beyond a purely biomedical pharmacological approach.

Finally, the accompanying instruction and narrative in the administration of OLPs is an important factor. In almost all OLP trials the provider clearly explained the inactive and inert nature of the pill, (often called a ‘sugar pill’), followed by some positive statements about this kind of treatment based on the circumstances that the placebo effect has been found to be powerful, that many other patients have already benefited from a placebo, that the body can also respond automatically after taking an inert pill, that a positive attitude about the pill might help but is not necessary or that “taking the pills faithfully is critical”^21^ ^p.^ ^2^. In one more recent study, patients were even told that “A few studies have shown that placebos without deception can have beneficial effects”^28^ ^p.10^. Some non-clinical studies examined the OLP-effect, and its dependence on differing instructions, in an experimental setting^29,30^. The results of these two studies suggest that a narrative that might raise positive expectations in the participants plays a crucial role in eliciting OLP effects, although it is unclear whether these findings from experimental studies can be generalized to a clinical context.

Despite these methodological challenges, an assessment of the current state of research is crucial. In an earlier systematic review and meta-analysis, Charlesworth et al.^29^ summarized five RCTs and found a positive medium-sized effect over all studies for OLPs compared to no treatment (NT) [standardized mean difference (SMD) = 0.88]. Their literature search was conducted in 2015 and there have been several trials published since then. Additionally, the validity of that meta-analysis is comparatively low due to the limited amount of studies and the moderate risk of bias^29^. The research on OLPs is still in its infancy and most of the studies have small sample sizes and short durations^24^. Nevertheless, the amount of studies in this field is growing. There are several recent reviews of OLP research^22–25^ but none of them are systematic. Thus, a systematic overview of the current state of research is missing in the area of OLPs. The aim of the present study is to assess, through a systematic review and meta-analysis, whether the treatment of patients with OLPs is significantly more effective than NT (or treatment as usual (TAU), if both groups are treated) in different intervention forms, patient conditions and outcomes. Additionally, we plan to assess whether the instruction that is given with OLPs is related to their efficacy. Based on this theoretical background the following hypotheses are proposed: (1) treatment with OLPs is more effective than no treatment, and (2) positive instructions increase the effectiveness of OLPs compared to no instruction. Our systematic review was preregistered with the International Prospective Register of Systematic Reviews (PROSPERO) 2020 CRD42020161696.

## Results

### Study selection

**Figure 1:**
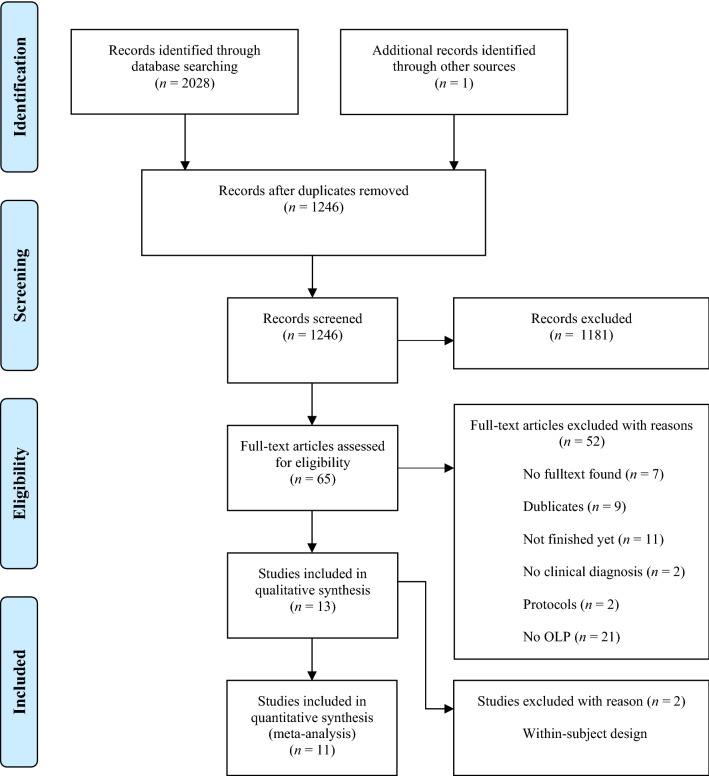
PRISMA flow chart for study selection.

The electronic database search identified 2028 citations (Table 1). After removing duplicates, we screened 1246 titles, 313 abstracts and 41 full text articles. Thirteen studies (834 participants) met all of the eligibility criteria and were included into the review. Due to a within-subject design, two studies were excluded from the meta-analysis. A flow chart detailing the process of study identification and selection is shown in Fig. 1. Tables with the characteristics of included studies (Table 2) are shown below. The detailed description of interventions is displayed in Table 3.

**Table 1:** Electronic databases searched and number of results.

Database	Papers identified
Embase (1947—February 2020)	434
MEDLINE (1966—February 2020)	279
PsycINFO (1967—February 2020)	383
CENTRAL (no inception date—February 2020)	932
Total	2028

**Table 2:** Characteristics of included studies.

Title	Author	Country	Year	Condition	N	Female sex. %	Mean age, years	Treatment	Control treatment	Duration	Number of prim. outcomes	Primary outcome used for meta-analysis	Rational for choice
Open-label placebo treatment in chronic low back pain: a randomized controlled trial	Carvalho et al.^30^	Portugal	2016	Chronic low back pain	83	71	44	OLP (n = 41)	Treatment as usual (n = 42)	21 days	2	Roland Morris Disability Questionnaire	Most clinically relevant
Open-Label Placebo Treatment for Cancer-Related Fatigue: A Randomized-Controlled Clinical Trial	Hoenemeyer et al.^31^	US	2018	Cancer-related fatigue	74	67	57	OLP (n = 39)	Treatment as usual (n = 35)	21 days	2	Fatigue Symptom Inventory (FSI-14)	Most clinically relvant
Altered Placebo and Drug Labeling Changes the Outcome of Episodic Migraine Attacks	Kam-Hansen et al.^32^	US	2014	Migraine attacks	66	85	41	OLP (n = 66)	No treatment (n = 66) (within subject)	7 migraine attacks	1	Change in headache, pain score	Only primary outcome
Placebos without Deception: A Randomized Controlled Trial in Irritable Bowel Syndrome	Kaptchuk et al.^21^	US	2010	Irritable bowel syndrome	80	70	47	OLP (n = 37)	No treatment (n = 43)	21 days	1	IBSa Global Improvement Scale	Only primary outcome
Open-Label Placebo for Major Depressive Disorder: A Pilot Randomized Controlled Trial	Kelley et al.^37^	US	2012	Major depressive disorder	20	70	39	OLP (n = 11)	Waitlist (n = 9)	14 days	1	17-item Hamilton scale for depression	Only primary outcome
Effects of open-label placebo on pain, functional disability and spine mobility in chronic back pain patients: a randomized controlled trial	Kleine-Borgmann et al.^33^	Germany	2019	Chronic back pain	122	72	59	OLP (n = 63)	Treatment as usual (n = 59)	21 days	1	Composite pain intensity score 0–10	Only primary outcome
Open-Label placebo for the treatment of unipolar depression: Results from a randomized controlled trial. (in press)	Nitzan et al.^34^	Israel	2020	Major depressive disorder	38	74	50	OLP (n = 18)	Treatment as usual (n = 20)	28 days	1	QIDS	Only primary outcome
Open-label placebos for menopausal hot flushes—a randomized controlled trial	Pan et al.^28^	Germany	2020	Menopausal hot flushes	100	100	55	OLP (*n* = 50)	No treatment (*n* = 50)	4 weeks	2	Hot flush score	Most clinically relevant
Conditioned Placebo Dose Reduction: A new treatment in ADHD?	Sandler et al.^19^	US	2010	ADHD	93	22	10	Dose reduced/Placebo (*n* = 33)	Reduced Dose (*n* = 29), Full Dose (*n* = 31)^a^	8 weeks	1	IOWA Connoers Rating Scale (Parent Version)	Only primary outcome (parents closer to child)
Open-label use of placebos in the treatment of ADHD: a pilot study	Sandler and Bodfish^20^	US	2008	ADHD	26	27	Not stated (range 7 to 15)	OLP (*n* = 26)	50% of baseline medication (*n* = 26) (cross-over design)	21 days	4	Seven-point clinical global impression	Includes different perspectives
Open-label placebos improve symptoms in allergic rhinitis: A randomized controlled trial	Schaefer et al.^38^	Germany	2016	Allergic Rhinitis	25	84	26	OLP (*n* = 12)	Treatment as usual (*n* = 13)	14 days	2	Symptoms (self-developed questionnaire)	Most clinically relevant
Why do open-label placebos work? A randomized controlled trial of an open-label placebo induction with and without extended information about the placebo effect in allergic rhinitis	Schaefer et al.^35^	Germany	2018	Allergic Rhinitis	46	80	25	OLP with briefing (*n* = 13) OLP without briefing (*n* = 13)	TAU + (*n* = 9), TAU- (*n* = 11)	14 days	2	Symptoms (self-developed questionnaire)	Most clinically relevant
Open-label placebo reduces fatigue in cancer survivors: a randomized trial	Zhou et al.^36^	China	2019	Cancer-Related Fatigue	40	92	47	OLP (*n* = 20)	No treatment (*n* = 20)	21 days	1	Functional Assessment of Chronic Illness Therapy-Fatique (FACIT-F)	Only primary outcome

*IBS* irritable bowel syndrom.

^a^“Full dose group” was excluded. We only compared the “reduced dose” group to the “reduced dose + placebo” group, which was scored as TAU.

**Table 3:** Detailed description of interventions.

Study	Open label placebo	Verbal instruction		Procedure of OLP aministration: (1) No. of interactions (2) Length of interaction(s) (3) Provider and assessor interactions
Carvalho et al. 2016^30^	“A typical prescribed medicine bottle of placebo pills with a label clearly marked “placebo pills” and “take 2 pills twice a day.” The placebo pills were Swedish Orange gelatin capsules filled with microcrystalline cellulose, a common inert excipient for pharmaceuticals”	“The PI explained that the placebo pill was an inactive substance, like a flour pill, that contained no active medication in it. After informed consent, all participants were asked if they had heard of the “placebo effect” and explained in an approximately 15-min a priori script, adopted from an earlier OLP study,18 the following “4 discussion points”: (1) the placebe effect can be powerful, (2) the body automatically can respond to taking placebo pills like Pavlov dogs who salivated when they heard a bell, (3) a positive attitude can be helpful but is not necessary, and (4) taking the pills faithfully for the 21 days is critical. All participants were also shown a video clip (1 min 25 s) of a television news report, in which participants in an OLP trial of irritable bowel syndrome were interviewed”		(1) 3 visits (2) 15 min a priori script; 10–15 min midpoint visit (3) Blinded nurse; interaction with unblinded Pl at midpoint
Hoenemeyer et al. 2018^31^	“The Pl provided pre-packaged pills, clearly labeled “placebos”, along with oral and written instructions to take 2 placebo pills twice per day.”	“The 4 points were (1) placebo effects are powerful in double-blinded clinical trials and there is some evidence that placebos work even when patients know it’s placebos but we don’t know if they work when honestly prescribed for CRF; (2) placebo responses may be attributed to conditioning, expectancy and biological (e.g., neurological, genetic) factors; (3) an open mind is helpful but unrelated to outcomes that may happen automatically; and, (4) taking the placebo pills as prescribed for 21 days is important (OLP group).”		(1) 2 visits + 1 phone call at midpoint (2) Not reported (3) Blinded research assistants and blinded research specialists; interaction with unblinded Pl (an oncology behaviour specialist) at 2 visits * phone call
Kam-Hansen et al. 2014^32^	“Study drug envelope was labeled “placebo”.”	“Take pill 30 min after migraine onset”		(1) Not reported (2) Not reported (3) No reported interactions; patient self-report
Kaptchuk et al. 2010^21^	“Placebo pills were blue and maroon gelatin capsules filled with Avicel, a common inert excipient for pharmaceuticals”	“The provider clearly explained that the placebo pill was an inactive (i.e., ‘‘inert’’) substance like a sugar pill that contained no medication and then explained in an approximately fifteen minute a priori script the following ‘‘four discussion points:’’ (1) the placebo effect is powerful, (2) the body can automatically respond to taking placebo pills like Pavlov’s dogs who salivated when they heard a bell, (3) a positive attitude helps but is not necessary, and (4) taking the pills faithfully is critical.”		(1) 3 visits (2) 30 min initial interview process; 15 min midpoint (3) Blinded assessors; midpoint interaction with unblinded physician Pl
Kelley et al. 2012^37^	“A visually distinctive placebo capsule”; “Blue capsules containing microcrystalline cellulose.”	“The rationale included four points: (a) in RCTs placebos are roughly 80% as effective as antidepressants; (b) classical conditioning is a possible mechanism for automatic self-healing; (c) placebo-treated patients who are more compliant have better outcomes, therefore the placebos should be taken faithfully; and (d) positive expectations increase placebo effects, but it is OK to have doubts. Although the rationale was scripted, treating psychiatrists were encouraged to deliver the rationale in a natural and supportive manner that allowed for questions and answers.”		(1) 3 visits, 1 blinded (2) Not reported (3) Blinded clinicians
Kleine-Borgmann et al. 2019^33^	“Open-label placebo capsules containing microcrystalline cellulose twice a day for 21 days”	“All patients were shown a video providing standardized information about the placebo effect in general and recent research findings on potential beneficial effects of open-label placebo application.”		(1) 3 visits (2) Not reported (3) Blinded examiner
Nitzan et al. 2020^34^	“Each participant received two packets containing a one month supply of placebo (120 capsules) and was instructed to take two capsules in the morning and two in the evening.”	“There will be no active component in the tablet, but there is a good chance that it will alleviate some of the depressive symptoms. Furthermore, recent scientific evidence suggests that placebo tablets can be helpful in treating various medical conditions even if a person knows they are placebos”		(1) 3 visits (2) Not reported (3) Research team-member
Pan et al. 2020^28^	“The OLP group received a glass bottle containing the pills labelled “Placebos for menopausal hot flushes” with the original medication leaflet.”	“Each patient receives the following information about placebos: (1) the placebo effect is powerful; patients reported symptom improvement after taking placebos in double-blind drug trials, including hot flush trials. However, these participants were unaware of whether they were receiving a placebo or medicine. (2) A few studies have shown that placebos without deception can have beneficial effects. The body may react to the pill intake automatically (Depending on the patient’s prior knowledge, an example is given, e.g., the Pavlov dog or food poisoning for which certain foods cause queasiness and nausea). Being positive and believing in a positive effect can help but is not necessary. (4) Taking the pills faithfully twice a day is crucial since the custom of pill intake can contribute to the effect. Also, to the adhering instructions is important to maintain the quality of the study, i.e., if some patients would only take half their pills, we would be comparing several subgroups with the no-treatment group. (5) Finally, we do not know whether placebos without deception can reduce hot flushes. Therefore, we encourage patients to simply “give it a try.”.”		(1) 4 visits (2) Relevant biopsychosocial information 10–15 min (3) Assessments by blinded research assistant; clinical consultation with unblinded clinician at each visit
Sandler et al. 2010^19^	A “visually distinctive capsule “	“Children and parents were told explicitly at the beginning of the study that the inert capsule was a placebo that contained no active pharmaceutical ingredients. Also, they were told the study was designed to determine *if* the procedure was effective. In keeping with a proof of concept study, we were neutral regarding the likelihood of success. Positive expectancy was maintained, however, by referring to the placebo both as a *placebo* and as a *Dose Extender*. If either the child or parent raised questions about possible mechanisms of placebo effects, we briefly discussed possibilities of mind–body interactions, expectancy and conditioning (described as “a kind of learning”).”		(1) Not reported (2) Not reported; children randomised to the OLP group had an additional discussion of the placebo with the study physician (3) Interaction with physician Pl; assessments by unblinded parents and blinded school teachers
Sandler and Bodfish 2008^20^	“A visually distinctive placebo capsule”	“This little capsule is a placebo. Placebos have been used a lot in treating people. It is called ‘Dose Extender’. As you can see, it’s different from __ *(give name of prescribed stimulant)*. Dose Extender is something new. It has no drug in it. I can promise you that it won’t hurt you at all. It has no real side effects. But it may help you to help yourself. It may work well with your, kind of like a booster to the dose of. That’s why it’s called a Dose Extender.”		(1) Not reported (2) Not reported (3) Interaction with unblinded physician Pl; assessments by unblinded parents and blinded schoolteachers
Schaefer et al. 2016^38^	“Participants in the placebo group received a white tube containing 28 placebo pills. The tube was labeled with the logo of the local university and the following information: ‘placebo pills (28), take one in the morning and one before night for 14 days	“We explained that placebos are inactive substances and that they contain no medications. Participants were further told that although placebos contain no medication, placebo effects may still be powerful. The effect was explained to them by pointing out that the body may automatically respond to taking placebo pills, like Pavlov’s dogs that salivated when they heard the bell. In addition, they were told that a positive attitude may be helpful for the placebo effect, but is not necessary. Last, they were told that those participants who were in the placebo group needed to take the placebos faithfully		(1) 2 visits (2) Not reported (3) Not reported, nor wether blinded
Schaefer et al. 2018^35^	See above (Schaefer et al., 2016)	With briefing: See above (Schaefer et al., 2016)	Without briefing: “placebos were explained as containing no medications, similar to a sugar pill.”	(1) 2 visits (2) Not reported (3) Experimenter
Zhou et al. 2018^36^	“Placebos were small red tablets containing microcrystalline cellulose, FD&C Red 40 and ethyl alcohol, manufactured and labeled by an FDA-registered pharmacy.”	“Following a written script, investigators described the study rationale, possible impact of placebo on CRF, prior evidence of the impact of placebo on symptoms including fatigue, and answered participants’ questions”		(1) 1 visit + 2 phone calls (2) Not reported (3) Research assistants

### Risk of bias

**Figure 2:**
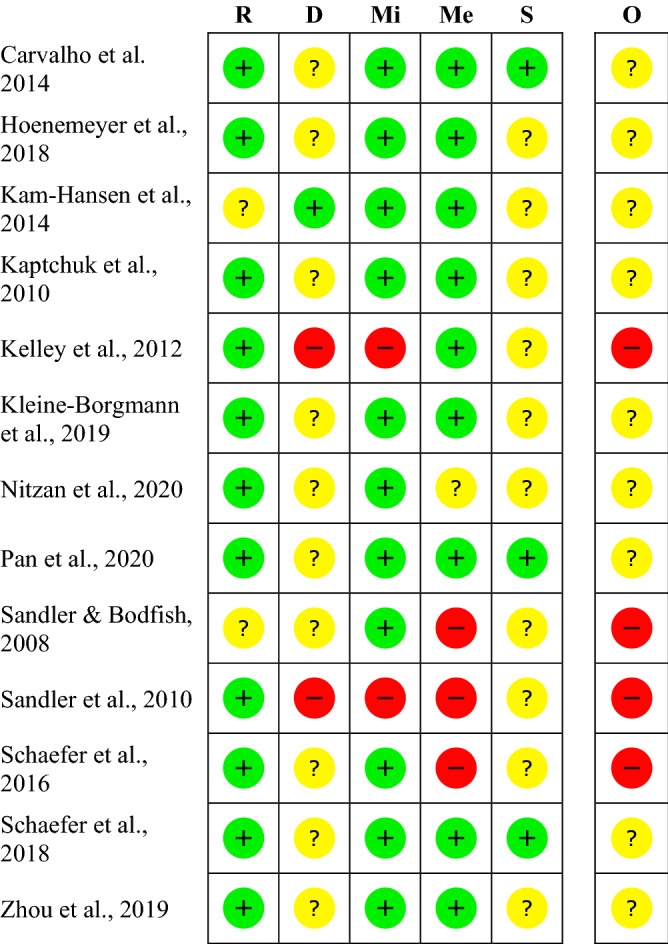
Within studies ‘risk of bias assessment for RCT on five ROB 2 criteria and overall. The risk of bias evaluation in the categories: bias arising from the randomization process (R), bias due to deviations from intended interventions (D), bias due to missing outcome data (Mi), bias in measurement of the outcome (Me), bias in selection of the reported result (S); overall risk of bias (O). Red symbols: high risk of bias; yellow symbols: some concerns; green symbols: low risk of bias.

The risk of bias varies between the studies. The rating of most studies (69%) resulted in “some concerns”^28,30–36^. Four studies (31%) were found to have a “high risk of bias”^19,20,37,38^ (see Fig. 2). Per definition, none of the participants was blinded to the treatment, so we did not rate this as a risk. When carers and people delivering the intervention were not blinded, we rated the risk with “some concerns”, as the risk of a different handling of patients depending on the group-membership cannot be ruled out. The high risk is mostly due to a considerable amount of missing outcome data and unblinded outcome assessors.

### Publication bias

**Figure 3:**
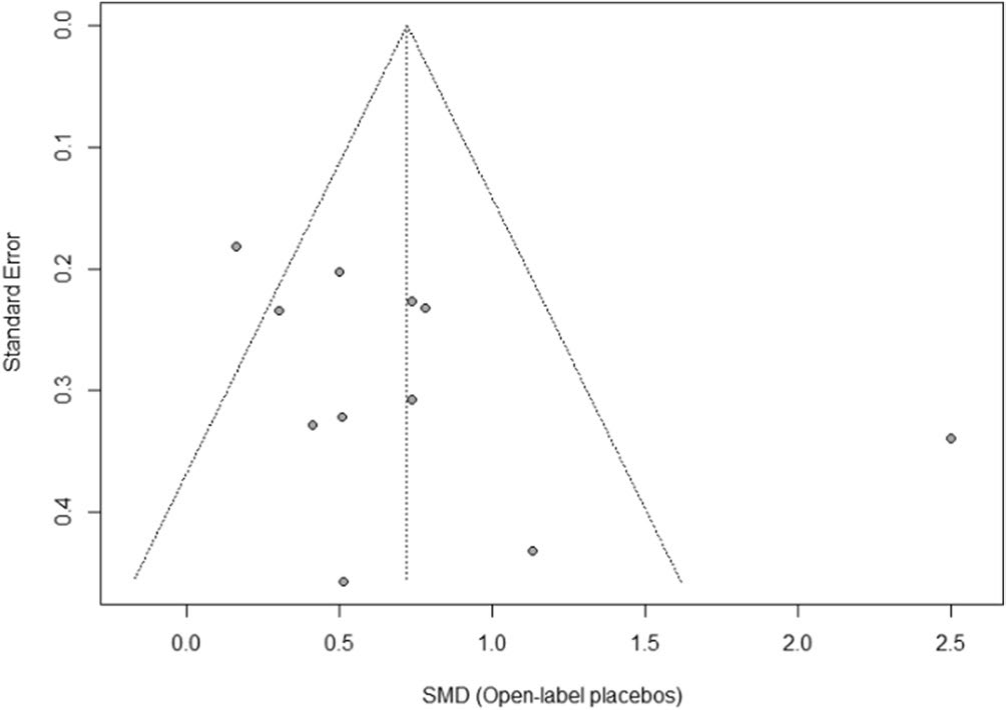
Funnel plot of standardized between-group OLP vs. NT scores. Funnel plot of standardized mean difference (SMD) vs. standard error. The dotted lines indicate the triangular region within which 95% of studies are expected to lie in the absence of publication bias.

The funnel plot displaying SMDs and the respective standard error for each RCT can be seen in Fig. 3. It shows signs of asymmetry. But the Egger’s regression test does not indicate a statistically significant departure from symmetry (intercept 3.44, 95% CI -0.71 – 7.59, *p* = 0.14). Thus, the risk of publication bias is limited. Nevertheless, the small number of studies (the “small-study effect”, affected by substantial heterogeneity, small samples, short duration, and partially high risk of bias^36^) may increase the risk of publication bias. The risk for the so-called “time lag bias” is also comparatively high, due to the early state of research in this field. This bias indicated that trials with negative results are published with some delay^39^.

### Synthesis of results

We included k = 11 studies (N = 654 participants) into the meta-analysis. Two studies were excluded because they have a within-subject design. Due to the exclusion of the full dose group in the study from Sandler et al.^19^, the number of participants was further reduced by *N* = 31. The test on heterogeneity is significant [χ^2^ (df = 10) = 41.14, *p* = 0.0001, *I*^2^ = 76%], demonstrating some additional variance.

**Figure 4:**
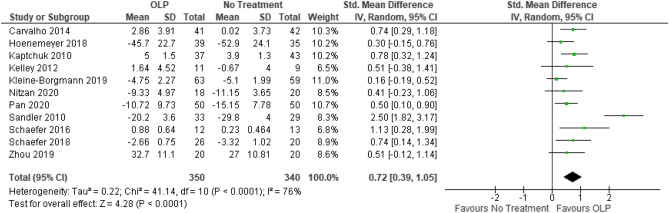
Forest plot for main outcome. Studies with open-label placebo (OLP) group and no treatment group were weighted using sample size (Total), means and standard deviations (SD). The means are shown by the green squares and the whiskers are representing the 95% confidence interval (CI). Overall standardized mean difference was calculated using the random effects model.

We found a significant positive effect of OLPs compared to no (additional) treatment SMD = 0.72, 95% CI 0.39–1.05, *p* < 0.0001 (Fig. 4).

### Additional analysis

For an explorative sensitivity analysis, we excluded four studies with a high risk of bias^19,20,37,38^ in order to obtain a best-evidence synthesis. In this sample the heterogeneity decreased to a non-significant level [χ^2^ (df = 7) = 7.32, *p* = 0.30, *I*^2^ = 4%] and almost all variance could be explained by a sampling error. The corresponding effect size was lower, but still significant (SMD = 0.49, 95% CI 0.32–0.66, *p* < 0.00001).

We also assessed exploratory whether there is a difference in control conditions. Overall, seven trials had TAU as control group, three trials had ‘no treatment at all’ as control condition and one trial had a wait-list design (see also Table 2). TAU controlled trials resulted in SMD = 0.82 (95% CI 0.29–1.34, *p* < 0.002) with a somewhat larger heterogeneity of *I*^2^ = 85% (χ^2^ = 40.15, *df* = 6, *p* < 0.00001). The no treatment at all trials resulted in SMD = 0.60 (95% CI 0.33–0.87, *p* < 0.0001) with no heterogeneity of *I*^2^ = 0% (χ^2^ = 0.92, *df* = 2, *p* = 0.63).

Furthermore, we conducted a sub-group analysis in which the two studies that were not yet peer-reviewed at the date of inclusion were excluded. This analysis yielded a slightly larger SMD with about the same heterogeneity: SMD = 0.79 (95 CI 0.38–1.20, *p* < 0.0002), *I*^2^ = 80% (χ^2^ = 40.28, *df* = 8, *p* < 0.00001).

### Results of individual studies

Carvalho et al.^30^ tested two randomized groups of patients (*N* = 83) with chronic lower back pain. They received either no additional treatment (TAU) or OLPs for 21 days. The investigators measured two primary outcomes, pain intensity and back-related dysfunction assessed by the Roland Morris Disability Questionnaire. At baseline, the TAU group reported lower baseline minimum pain scores, but there were no other significant differences. After 3 weeks, the OLP group had a significantly reduced disability (*p* < 0.001) and significantly reduced pain (*p* < 0.001).

Hoenemeyer et al.^31^ carried out a 21-day RCT with two groups to examine whether OLPs reduce fatigue in cancer survivors (*N* = 73). The OLP group (*n* = 39) received placebo pills while the control group (*n* = 35) received no additional treatment (TAU). The primary outcome fatigue was assessed via the Fatigue Symptom Inventory (FSI-14) and the Multidimensional Fatigue Symptom Inventory Short Form (MFSI-SF30). The difference between the groups concerning fatigue symptoms was significant after 21 days according to FSI-14 (*p* = 0.008) and MFSI-SF30 (*p* = 0.002).

Kam-Hansen et al.^32^ did a randomized study comparing the efficacy of two treatments (placebo/maxalt and no treatment/baseline) along with three different types of information (positive/negative/unclear) in patients with migraine-attacks (*N* = 66) using a within-subject design. The outcome was a pain scale from 0–10 two hours after treatment. The pain scores after taking an OLP-pill were significantly lower than those after no treatment (*p* = 0.001).

Kaptchuk et al.^21^ carried out a RCT with 80 patients suffering from irritable bowel syndrome. One group received OLPs for 21 days, the other group got NT. Differences were measured with the IBS Global Improvement Scale after 3 weeks. The OLP group experienced a significantly higher improvement than the control group (*p* < 0.002).

Kelley et al.^37^ did a pilot study with two parallel groups examining the OLP effect in patients with Major Depressive Disorder (*N* = 20). The OLP group received placebo capsules for 14 days, while the other group was on the wait-list. The primary outcome was the 17-item Hamilton scale for depression. They found no significant difference between the groups after the treatment (*p* = 0.26).

Kleine-Borgmann et al.^33^ tested the efficacy of OLPs in patients with chronic back pain in a RCT. One group (*n* = 63) received OLPs for 21 days and the other group (*n* = 59) had no additional treatment (TAU). Changes were measured with a composite pain score from 0–10. Improvement was significant for the OLP group compared to the control group (*p* = 0.001).

Nitzan et al.^34^ examined whether OLP-treatment is different than TAU in the context of therapy for depression in a parallel-group design. The intervention group (*n* = 18) received OLP-pills for 4 weeks, while the control group (*n* = 20) got no additional treatment. The primary outcome was the self-report questionnaire for assessment of depressive symptoms (QIDS). Measures taken at the endpoint of the trial showed no significant difference between the groups (*p* = 0.203).

Pan et al.^28^ carried out a RCT, which tested 100 patients with menopausal hot flushes. One group (*n* = 50) received OLPs for 4 weeks, and the other group (*n* = 50) got no treatment (NT). After 4 weeks, the OLP group was randomized again into two groups. One received OLPs for another 4 weeks, while the other group got NT. The primary outcome was a hot flush composite score (frequency x intensity). The other primary outcome was change in problem rating measured with the Hot Flush Rating Scale (HFRS). After 4 weeks of OLP treatment, hot flushes were significantly reduced compared to the control group (*p* < 0.001). The HFRS did not change (*p* = 0.24).

Sandler and Bodfish^20^ tested if conditioned OLPs have an effect as dose-extender on children with ADHD in a pilot RCT. Participants (*N* = 26) were randomized into two groups and both received a full dose of stimulant medication for one week. Then they received in a cross-over design either (1) a 50% dose in the second week and a 50% dose + open label placebos in the third week, or (2) the reversed order. The most clinical outcome was the Clinical Global Impression scale (CGI) which was completed by the study physician after interviewing the children, the parents, and the blinded teachers. The placebos had a significant effect as a dose extender (*p* = 0.004). Other primary outcomes were the IOWA Conners ADHD rating scale (P-IOWA), the Pittsburgh side effects rating scale (PSERS) and the teacher version of the IOWA Conners ADHD rating scale (T-IOWA). The results of the comparison between the 50% dose and the 50% + OLP condition were not reported.

Sandler et al.^19^ examined whether conditioned OLPs have an effect on children with ADHD. They were randomized into three groups. One group (*n* = 31) received a full dose (FD) of stimulant medication for two months. Another group (*n* = 29) received the full dose for one month and a reduced dose (RD) for another month. The third group (*n* = 33) also received a full dose for one month with additional placebos, and a reduced dose in the second month with additional placebos (RD/P). The primary outcome was the IOWA Connoers-Rating Scale (parent version). After 8 weeks, the RD group deteriorated significantly compared to the RD/P group (*p* = 0.0004), according to the unblinded parents.

Schaefer et al.^38^ tested the efficacy of OLPs in patients with allergic rhinitis. Participants (*N* = 25) were randomized into two groups. The OLP group was treated for 14 days with inert placebo pills, while the control group received no additional treatment (TAU). The primary outcome was a self-developed symptoms questionnaire. After the treatment, the OLP group had significantly fewer symptoms than the control group (*p* = 0.05). The second primary outcome was the SF-36, which examines the quality of life (*p* = 0.45).

Schaefer et al.^35^ conducted a RCT with four groups of patients with allergic rhinitis in order to test if the briefing combined with the OLP treatment is a significant factor for OLP effects. Two OLP groups, one with briefing (*n* = 13) and one without briefing (*n* = 13) were compared to two control groups, that received no additional treatment (TAU) either with (*n* = 9) or without briefing (*n* = 11). The primary outcome was again a self-developed symptoms questionnaire. The OLP treatment was significantly better than the TAU, independent of the briefing (*p* = 0.02). The SF-36 showed no significant change (no p-value reported).

Zhou et al.^36^ examined whether an OLP treatment had an effect on patients suffering from cancer-related fatigue. The 40 participants were randomized into two groups; the OLP group received OLP-pills for 21 days while the control group got NT. They used the Functional Assessment of Chronic Illness Therapy-Fatigue (FACIT-F) as a primary outcome. Patients who received OLPs significantly benefitted from the treatment (*p* = 0.02).

## Discussion

This systematic review and meta-analysis were conducted in order to get an overview of the current body of research, and to find a pooled effect-size estimate of OLPs. We found thirteen studies that met our eligibility criteria. Eleven of them assessed the effect of OLPs in patients compared to no treatment or treatment as usual in two separate groups, making them eligible for the meta-analysis.

The quantitative synthesis of these trials revealed a significant, medium-sized effect of OLPs across those eleven RCTs. All studies included into the meta-analysis examined the efficacy of the OLP treatment, providing an accompanying narrative. Therefore, we were not able to assess the role of the instruction accompanying the placebo administration. Consequently, our hypothesis, that a positive instruction increases the efficacy of OLPs compared to no instruction, could neither be supported nor proven wrong at this stage.

Regarding the interpretation of the overall effect size of SMD = 0.72 one needs to consider some limiting factors. First, we detected hints of a publication bias in the study sample, but the respective test was not significant. The quantitative basis of the meta-analysis is based on a small number of studies, reflecting the early state of research in this field. Moreover, the set of studies showed some heterogeneity. Finally, four studies were rated to have a high risk of bias, and nine to have some concerns.

In order to assess the impact of these high-risk studies we performed an exploratory best-evidence synthesis. We excluded the four studies with a high risk of bias. In this analysis, the heterogeneity could be reduced to a non-critical value and almost all variance in the set of studies could be explained by a sampling error (*I*^2^ = 4%). With the exclusion of these four studies the mean effect size was reduced to a more conservative SMD = 0.49.

Regardless of this reduction of the overall effect, the same conclusions about the treatment-effect of OLPs can be drawn, although the lack of robustness means that interpretations require some caution. The decrease of heterogeneity shows that methodological impairments might be responsible for the considerable unexplained variance in our results. We abstained from carrying out a further sensitivity analysis for explaining heterogeneity because of the small number of studies.

The positive effect of OLPs is in line with findings of the earlier meta-analysis that analyzed five studies (234 participants)^29^. The updated analysis includes thirteen studies (781 participants) in the systematic review and eleven studies (654 participants) in the meta-analysis and thus, considerably broadens the database. The inclusion of more studies, some of which analyzed larger sample sizes, allows for a higher certainty of the overall OLP effect. We also included one RCT^19^, published in 2010, that was not included by Charleston et al.^29^ even though it should have met their eligibility criteria. This indicates a more thorough searching strategy in the present study. Moreover, Charlesworth et al.^29^ included a within-subject trial^20^ in their meta-analysis without applying a different effect-size formula suitable for this type of study design^37^. By excluding this study from our meta-analysis, our overall effect provides further certainty. We included a second study, published in 2014^32^, in our review which was not included by Charlesworth et al.^31^ even though it provides an appropriate comparison of the OLP and NT condition. We excluded it from our meta-analysis because of the within-subject design. In summary, the present review and meta-analysis should result in a more reliable picture of the current body of research on OLPs.

In summarizing OLP trials, we need to also consider different control conditions. In our study sample control conditions for OLP arms were either *TAU*, ‘*no treatment at all*’ or *wait-list*. The overall sample was too small to perform sensitivity analyses. Overall TAU controlled trials had slightly larger effect sizes than ‘no treatment at all trials’. For ‘no treatment at all’ trials heterogeneity dropped to zero, indicating a homogenous sub-group. However, one needs to consider that the analysis of TAU trials is confounded with high risk of bias trials. Overall, the choice, type and exact definition of control conditions are open issues in OLP research.

The overall effect of OLPs compared to NT is very promising. The effect of *deceptive* placebos is estimated as SMD = 0.23 (95% CI 0.17–0.28) in a much larger meta-analysis including 158 trials with more than 10,000 patients^40^. It is doubtful whether this effect size can be compared to the present finding since the meta-analysis by Hróbjartsson and Gøtzsche^40^ included a wide range of conditions, including many which are not expected to respond to placebo. Another reason for this comparably larger effect in OLPs could be that in an early state of research, “positive” studies are more likely to be published (time-lag bias). Furthermore, OLPs might produce some additional effects beyond classical conditioning and expectations. Because of the novelty of this kind of treatment, patients seemed to enjoy the treatment and described it as “crazy” according to the intake and exit interviews^23,41^. Many patients were frustrated by multiple unsuccessful treatments and chose this counterintuitive intervention from a state of despair^24^, which may have produced new hope after the previous psychological strain. The patients were, by the very definition of the *open-label* placebo, not blinded, and all outcomes were self-reported, which facilitates the impact of these factors. According to Ongaro and Kaptchuk^42^, the contradictory messages embedded in the provided narrative “this placebo pill may help; it’s an inert pill without physiological effect” can produce a cognitive dissonance, which disturbs central sensitization. This effect is based on the idea of the so called “Bayesian Brain”, which creates a prediction-driven perception of the world^23,42^. The research around this field suggests that the perception of body sensations and the environment is cognitively modulated by expectations rather than “a bottom-up readout of sensory signals”^42^ ^p.1^. The cognitive dissonance due to the OLP instruction might alter the familiar interpretation of symptoms and can cause a less intense sensation. A similar effect could be also shown empirically in an active placebo study^43^. The extent to which the decrease of central sensitization is actually affecting measurable health-related symptoms should be examined in OLP studies with objective outcomes. Also, further research on the distinguishing features of deceptive and open placebos is required.

With respect to the small number of studies included, there are some features that should be noted. The research in OLPs to date has been carried out by only a few authors. Some authors are therefore involved in several of the included studies, which reduces the independence of the different trials. It would be preferable if more independent replications were conducted in the future.

Sandler et al.^19,20^ tested children, while all other studies tested adults. Children are more suggestible and have a higher placebo response than adults in the context of trials with patients suffering from mental disorders^44^. Furthermore, they administered OLPs as “dose extenders”. This procedure draws mainly on placebo effects due to classical conditioning and is different from all other studies in the meta-analysis, which are expectancy-based. The differences between these two approaches to OLP effects would be an interesting subject to address in future meta-analyses on larger databases.

We also included the study of Pan et al.^28^, even though the diagnosis for menopausal hot flushes was not validated by gynecologists. The patients filled out a screening questionnaire and were seen by a psychologist who confirmed the diagnosis during the study. According to the respective guidelines^45^ most practitioners rely on women´s self-reports in the treatment of menopausal hot flushes. Hormone tests are not primarily recommended.

Even though this study shows the promising potential of OLP treatments, the overall pooled effect estimate gives only a broad hint of the real effectiveness of OLPs. Due to the short history of this field of research and the small amount of studies, we are still far from being able to understand the full implications for clinical decisions. Even though there are positive findings for OLPs in a range of physical and mental conditions (back pain, migraine, cancer-related fatigue, ADHD, allergic rhinitis and irritable bowel syndrome), most of the studies still have small sample sizes and a short duration of treatment and follow-up measures.

Additionally, all primary outcomes that were included into the meta-analysis are based on self-reports. Subjective outcomes can be biased (e.g. wishing to please the examiner). Due to the lack of blinding, patients knew about their treatment and it is difficult to interpret if the impact of this knowledge is relevant. This applies especially for the control group, whose reports could be influenced by disappointment. As mentioned before, these effects are not clearly differentiated from placebo and nocebo effect. However, all studies examined conditions (major depression, cancer-related fatigue, pain, menopausal hot flushes, irritable bowel syndrome, ADHD, allergic rhinitis), which are mostly diagnosed and rated by self-reports. Only one study^33^ measured an objective outcome of mobility parameters in chronic lower back pain that demonstrated no significant effects while the self-reports did show significant subjective changes through OLPs. Another study included a questionnaire as an outcome, which was completed by blinded teachers^19^. The results were also not significant. Further research with objective outcomes and objective diagnostic tools in OLP treatment would be recommended to draw further conclusions about the measurable extent of OLP effects.

As the treatment with OLP is not blinded per definition, the advert for the recruitment of participants spoke of a “novel body-mind” treatment, which probably attracted only participants that were willing to try this kind of treatment. Such a self-selection lowers the generalizability of the results for all patients with the same condition, but open-label studies always need to deal with this selection bias.

This systematic review and meta-analysis were conducted in an early state of the research of OLPs. Therefore, we examined the intervention on a meta-level based on studies measuring different conditions, and thus different outcomes were combined. Since there is no appropriate method for these kinds of systematic reviews we followed the methodological approach of the PICO philosophy^46^. These guidelines (e.g. Cochrane) are aligned to examine a specific population with *one* condition and *one* outcome. Therefore, conceptualizations like ROB 2 needed to be adjusted to our studies, which lowered the informative value. The studies are maybe too different to be compared with the common methods, which reduces the reliability of the effect. Future clinical research on meta-level interventions would benefit from appropriate methods. Independently of this, a meta-analysis of OLPs would be desirable for studies based on the same condition and same outcome. At the moment, such a meta-analysis would include a maximum of two studies, which does not result in a reliable picture.

Our study has some limitations. We did not explicitly search for grey literature, like unpublished but completed studies, dissertations, and conference abstracts. This limitation may have led to a potential publication bias of the included studies. However, the database research provided not only results from published studies, but also registered trials that were still ongoing or never finished or published. The authors of potentially eligible titles were contacted via e-mail, which allowed us to include two completed trials that were under review. In the meantime, both of these studies have been published. We made a sub-group analysis of only the studies published before the inclusion date, which revealed no substantial differences. Another limitation is that we were not able to find evidence regarding the role of the instruction accompanying the placebo administration, since this was assessed in only one trial. Future studies should take into account whether the instruction influences the treatment effect of OLPs in the clinical context. The role of suggestion prior to the treatment should be examined, especially in contrast to an instruction that only contains the information that the pill is a placebo. It would also be interesting to study whether these suggestions actually influence already established expectations and also more general mindsets^47^. Generally, the role of expectations should be considered prior to the treatment, both in medical care and psychotherapy. A qualitative interview of patients receiving OLP treatments would provide further information about the patient’s attitude and the modes of actions in this treatment. Due to the lack of blinding of OLPs, positive expectations are particularly crucial.

The treatment with OLPs might have a significant effect. Patients that suffer from pain, allergic rhinitis, cancer-related fatigue, menopausal hot flushes, and ADHD benefited from the OLP treatment. OLPs might be as effective, or even more effective, than deceptive placebos. The current research in this field is not yet sufficient to adequately explain the responsible modes of action. More studies with a longer duration and more participants are required in this field, but the results of this study suggest a promising and novel treatment approach in the context of placebos. It also emphasizes the role and power of contextual factors in the treatment of patients such as narratives, instructions, expectations and interactions. OLP treatment takes the patient’s autonomy into account and addresses the self-healing process of the body. Beyond that, patients do not need to be blinded to their treatment, which allows them to be more aware of their conscious and unconscious reactions to the treatment. It also gives healthcare-providers the possibility to administer placebos without deception and thus, with fewer ethical concerns. However, this does not mean that the use of OLPs is free from ethical problems. Two recent publications^48,49^ have pointed to other ethical issues in the use of OLPs such as self-stigmatization, testimonial injustice and the risk of a medicalization of issues that are more socially or environmentally determined. Nevertheless, even if the current body of research on OLP treatments does not yet allow for clinical recommendations, it supports the conclusion that it is a promising approach that is worth pursuing.

## Methods

### Eligibility criteria

Studies were included if they were randomized controlled trials, which also includes certain within-subject designs as, for example, in cross-over trials. The control group or condition is defined as receiving either no treatment (NT) or treatment as usual (TAU) while TAU must have been the same in both groups. Patients needed to have a medical condition or mental disorder, diagnosed by a clinician or psychologist. Studies needed to provide the necessary information for effect size calculation. We did not apply language, age, or date restrictions.

We excluded studies, which tested participants with a condition that was only diagnosed by self-report as well as studies with healthy volunteers.

### Information sources

On the 24th February 2020 we searched for studies using the databases EMBASE via Elsevier Medline via PubMed, PsycINFO via EBSCO, and The Cochrane Central Register of Controlled Trials (CENTRAL). We also screened the Journal of Interdisciplinary placebo Studies DATABASE (JIPS) and the Program in Placebo Studies & Therapeutic Encounter (PiPS). No additional search was done after February 2020. Nevertheless, we included studies that were found in our search but published later. After e-mail contact with two authors (Y. Pan, and U. Nitzan) they provided their submitted manuscripts.

### Search strategy

In order to update the review from Charlesworth et al.^29^ we used a similar search strategy. We additionally searched the database PsycINFO and expanded our search string for a more sensitive search. For the databases Medline and EMBASE we searched with less proximity operators. The search strategy for all databases is listed in the appendix (Tables S1 to S4).

### Study selection

After removing duplicates, two investigators independently screened all remaining titles, abstracts, and full records for eligibility. Differences in results were discussed between the investigators. In addition, a third person was consulted for two studies. The main reasons for exclusion were that the placebo arm was a control condition for a treatment arm in an open-label trial, the absence of a RCT or the fact that there were no clinical patients as participants. For some titles, no abstract or full texts were found. After sending requests via ResearchGate and e-mail to the authors, most studies were either not yet finished or no reply was received.

Only studies with independent groups for each treatment condition (OLP and no (added) treatment) were included into the meta-analysis. Two submitted manuscripts^28,34^ were provided by the authors, and in the meantime one trial was also published.

### Data extraction

We extracted data about the author, year, country of trial execution, duration of treatment, number of participants, exact intervention and control condition, number of primary outcomes and type of outcome used. Additionally, we extracted information about the exact verbal instruction that was given to the patients. Data extraction was done by two independent investigators. The means and standard deviations of the OLP and control condition, as well as the number of participants in each group were extracted. We defined the endpoint as the end of the OLP treatment. In some studies, the control-group also received OLPs after two weeks^37^ or after four weeks^34^. Due to the need for a NT control, we decided to choose the endpoint where the control group still received NT. We did not compare follow-up endpoint because of high heterogeneity between the study-designs. In one study^19^ we only compared the reduced dose group with the reduced dose/placebo group (detailed information in Table 1) because the full-dose group did not meet our eligibility criteria for the control group (NT).

We chose the primary outcome if there was only one reported (see Table 2). Other outcomes were not included in our meta-analysis. In several studies, two primary outcomes were reported. In most cases, this was a symptom-oriented assessment and a scale referring to disease-related quality of life. In these cases, we selected the most clinically relevant outcome. This was in all studies the relevant symptom-related scale, with one exception. In the trial on chronic back pain by Carvalho^30^ an unusual average of numerical rating scales was applied as the primary outcome in combination with the well-established and well-validated Roland–Morris Disability Questionnaire. Here the latter was selected as the more clinically relevant outcome.

### Risk of bias assessment

The risk of bias assessment of individual studies was performed by two independent reviewers. Discrepancies were resolved by an expert’s opinion. We used the revised Cochrane risk of bias tool (ROB 2) for randomized trials^50^. We evaluated biases that arose from the randomization process, deviations from intended interventions, missing outcome data, measurement of the outcome and selection of the reported result. We desisted from increasing the risk of bias due to the lack of blinding of participants for two reasons. First, patients in the OLP treatment cannot be blinded by definition. Second, the additional effect of knowing about the group allocation cannot be separated from the placebo or nocebo effect (excitement or disappointment respectively). Even if this may affect the results of the study, it cannot be rated as a bias because it is the object of investigation in our study.

### Risk of bias across studies

We did not conduct the risk of bias across studies using the GRADE (Grading of Recommendations, Assessment, Development and Evaluations) approach from Cochrane because it addresses the quality of evidence for the corporate outcome. The present study is conducted on a meta-level, which means that the studies have various outcomes and thus GRADE is not a suitable assessment. Therefore, we fell back on the PRISMA guidelines^51^ for addressing the risk of bias across studies.

We examined the possibility that the included trials are biased by availability (publication bias). Therefore, we investigated publication bias of the meta-analysis by plotting the effect by the inverse of its standard error and visualizing it in a funnel plot. To evaluate asymmetry, we used visual inspection as well as Egger’s regression test^52^.

The detection of *selective reporting bias* addresses those studies which are excluded as they do not provide sufficient information to compute effect sizes. None of our studies were excluded for this reason, which eliminates the risk of this bias.

### Statistical procedures

We conducted our meta-analysis in RevMan version 5.4^53^ using the random effects model according to the diversity of patients, study designs and outcomes. The computations of Egger's Regression test and the display of figure 3 were made with the packages *meta* and *dmetar* of the statistical software *R*. All studies reported continuous outcomes.

In the study by Sandler et al.^19^, we used only a subset of data, contrasting the reduced-dose condition (RD) and the reduced-dose/placebo condition (RD/P). Schaefer et al.^35^ randomized participants into four groups, using a 2 × 2 design, providing for each intervention (OLP or NT) either a positive briefing or not. For the meta-analysis, we used combined data for the OLP and NT groups.

We calculated the overall standardized mean-difference (SMD) by dividing the difference in mean outcome between the groups by the standard deviation of outcome among participants, and the 95% confidence interval (CI). Additionally, we took the heterogeneity between the studies into account, measuring the χ^2^ test *Cochran’s Q* and *Higgins I*^2^^51^ in order to determine the amount of unexplained variance. In order to adjust scales which increase or decrease with disease severity in the same direction, we multiplied the mean values with − 1 in some studies^19,28,31,33–35^ according to the Cochrane handbook^54^.

### Additional analyses

We planned to compare studies with and without a positive narrative in a sub-group analysis. However, the number of studies (k = 1) was not sufficient to examine this pre-specified analysis. Therefore, our hypothesis that the positive narrative provided increased the efficacy of the OLP treatment could not be tested.

We decided to undertake an explorative best-evidence synthesis by excluding studies with a high risk of bias. We further performed two exploratory sub-group analyses to compare different control conditions as well as peer-reviewed vs. not peer-reviewed trials.

## Supplementary Information

Supplementary Tables.

## Data Availability

All data from this study will be uploaded to a public repository upon acceptance of the manuscript for publication.
