# Author Correction: Effects of open-label placebos in clinical trials: a systematic review and meta-analysis

**DOI:** 10.1038/s41598-021-96604-0

**Published:** 2021-08-25

**Authors:** Melina von Wernsdorff, Martin Loef, Brunna Tuschen‑Caffier, Stefan Schmidt

**Affiliations:** 1 Department of Psychosomatic Medicine and Psychotherapy, Medical Center Freiburg, Faculty of Medicine, University of Freiburg, Hauptstr. 8, 79104 Freiburg, Germany; 2 Department of Psychology, Clinical Psychology and Psychotherapy, University of Freiburg, Freiburg, Germany; 3 CHS-Institut, Berlin, Germany; 4 Institute for Frontier Areas and Mental Health, Freiburg, Germany

Correction to: *Scientific Reports* 10.1038/s41598-021-83148-6, published online 16 February 2021

The original version of this Article contained errors, where the number of participants from Sandler et al.^19^ was incorrectly utilized.

Consequently, in the Results section, subheading “Publication bias”,

“The funnel plot displaying SMDs and the respective standard error for each RCT can be seen in Figure 3. It shows signs of substantial asymmetry. The Egger’s regression test also indicates a statistically significant departure from symmetry (intercept 3.33, 95% CI 0.54–6.13, *p* = 0.024), which means that there might be a considerable number of trials that were either not published or not identified by our search. Thus, a risk of publication bias can be assumed. Also the small number of studies (the “small-study effect”, affected by substantial heterogeneity, small samples, short duration, and partially high risk of bias^36^) increases the risk of publication bias. The risk for the so-called “time lag bias” is also comparatively high, due to the early state of research in this field. This bias indicated that trials with negative results are published with some delay^39^.”

now reads:

“The funnel plot displaying SMDs and the respective standard error for each RCT can be seen in Figure 3. It shows signs of asymmetry. But the Egger’s regression test does not indicate a statistically significant departure from symmetry (intercept 3.44, 95% CI − 0.71–7.59, *p* = 0.14). Thus, the risk of publication bias is limited. Nevertheless, the small number of studies (the “small-study effect”, affected by substantial heterogeneity, small samples, short duration, and partially high risk of bias^36^) may increase the risk of publication bias. The risk for the so-called “time lag bias” is also comparatively high, due to the early state of research in this field. This bias indicated that trials with negative results are published with some delay^39^.”

In Figure 3, the funnel plot still shows signs of asymmetry but the Egger’s regression test is not significant.

As a result, the Figure legend was incorrect.

The original Figure 3 and accompanying legend appear below.

**Figure 3:**
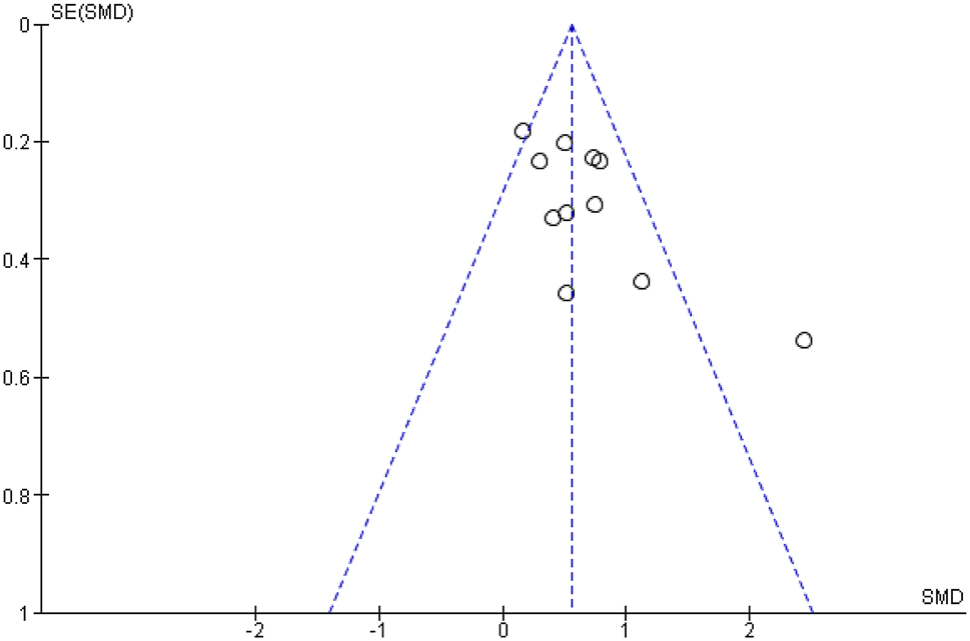
Funnel plot of standardized between-group OLP vs. NT scores. Funnel plot of standardized mean difference (SMD) vs. standard error (SE). The dotted lines indicate the triangular region within which 95% of studies are expected to lie in the absence of publication biases.

In the Discussion section,

“First, we detected hints of a publication bias in the study sample.”

now reads:

“First, we detected hints of a publication bias in the study sample, but the respective test was not significant.”

Under the same section,

“This limitation promotes a publication bias of the included studies. Nevertheless, the database research provided not only results from published studies, but also registered trials that were still ongoing or never finished or published.”

now reads:

“This limitation may have led to a potential publication bias of the included studies. However, the database research provided not only results from published studies, but also registered trials that were still ongoing or never finished or published.”

Lastly, under the Methods section, subheading “Statistical procedures”,

“We conducted our meta-analysis in RevMan version 5.4^53^ using the random effects model according to the diversity of patients, study designs and outcomes. All studies reported continuous outcomes”

now reads:

“We conducted our meta-analysis in RevMan version 5.4^53^ using the random effects model according to the diversity of patients, study designs and outcomes. The computations of Egger's Regression test and the display of Figure 3 were made with the packages *meta* and *dmetar* of the statistical software *R*. All studies reported continuous outcomes.”

The original Article has been corrected.

