# Supplementary material for: Effects of open-label placebos in clinical trials: a systematic review and meta-analysis
Source: Sci Rep. 2021 Feb 16;11:3855. doi: 10.1038/s41598-021-83148-6 (PMC7887232; doi:10.1038/s41598-021-83148-6)
Supplement: Supplementary file 1 — Supplementary Tables. [file 41598_2021_83148_MOESM1_ESM.docx]

**Effects of Open-Label Placebos in Clinical Trials –**

**A Systematic Review and Meta-Analysis**

Melina von Wernsdorff^1,2^, Martin Loef^3^, Brunna Tuschen-Caffier^2^, Stefan Schmidt^1,4,*^

^1^ Department of Psychosomatic Medicine and Psychotherapy, Medical Center– University of Freiburg, Faculty of Medicine, University of Freiburg, Germany

^2^ Department for Clinical Psychology and Psychotherapy, Institute of Psychology, University of Freiburg, Germany

^3^ CHS-Institut, Berlin, Germany

^4^ Institute for Frontier Areas and Mental Health, Freiburg, Germany

* Corresponding author:

Prof. Dr. phil. Stefan Schmidt

Department of Psychosomatic Medicine and Psychotherapy

Medical Center Freiburg – University of Freiburg

Hauptstr. 8, 79104 Freiburg

Germany

Tel. +49-761-270-69280

stefan.schmidt@uniklinik-freiburg.de

**Supplemental Material**

**Table S1**

*Search Strategy in The Cochrane Central Register of Controlled Trials (CENTRAL)*

| Step | Searches | Results |
| --- | --- | --- |
| #1 | (placebos):ti,ab,kw | 295919 |
| #2 | (placebo*):ti | 66994 |
| #3 | #1 OR #2 | 295999 |
| #4 | (told OR nondecept* OR “non decept*” OR nonconceal* OR “non conceal*” OR nonblind* OR “non blind*” OR “without deception” OR “without conceal*” OR “without blind*”):ti,ab,kw | 4115 |
| #5 | #3 AND #4 | 578 |
| #6 | ((open OR “open label”) NEAR/1 placebo*):ti,ab,kw | 377 |
| #7 | #5 OR #6 | 932 |

**Table S2**

*Search Strategy in EMBASE via Elsevier*

| Step | Searches | Results |
| --- | --- | --- |
| #1 | Placebo:ti,ab,kw | 301755 |
| #2 | Placebos:ti,ab,kw | 3113 |
| #3 | Placebo*:ti | 52031 |
| #4 | #2 OR #3 | 53786 |
| #5 | told OR nondecept* OR “non decept*” OR nonconceal* OR “non conceal*” OR nonblind* OR “non blind*” OR “without deception” OR “without conceal*” OR “without blind*”:ti,ab | 16607 |
| #6 | #4 AND #5 | 201 |
| #7 | (open OR “open label”) NEAR/1 placebo* | 252 |
| #8 | #6 OR #7 | 434 |

**Table S3**

*Search Strategy in Medline via PubMed*

| Step | Searches | Results |
| --- | --- | --- |
| #1 | Placebos [Title/Abstract] | 3317 |
| #2 | ((#1)) OR ((Placebos/tu)[Title/Abstract] | 5582 |
| #3 | (Placebos[Title/Abstract]) OR (Placebos/tu[Title/Abstract]) | 3389 |
| #4 | Placebo*[Title] | 36322 |
| #5 | (#3) OR (#4) | 38416 |
| #6 | ((((((((((told[Title/Abstract]) OR (nondecept*[Title/Abstract])) OR (“non decept*”[Title/Abstract])) OR (nonconceal*[Title/Abstract])) OR (“non conceal*”[Title/Abstract])) OR (nonblind*[Title/Abstract])) OR “non blind*”[Title/Abstract])) OR (“without deception” [Title/Abstract])) OR (“without conceal” [Title/Abstract])) OR (“without blind*”[Title/Abstract]) | 11882 |
| #7 | #5 AND #6 | 136 |
| #8 | (“open placebo*”[Title/Abstract]) OR (“open label placebo*”[Title/Abstract]) | 154 |
| #9 | #7 OR #8 | 279 |

**Table S4**

*Search Strategy in PsycINFO via EBSCO*

| Step | Searches | Results |
| --- | --- | --- |
| #1 | TI Placebos OR AB Placebos | 39581 |
| #2 | TI Placebo* | 7704 |
| #3 | TI Placebo* OR #1 | 39582 |
| #4 | TI (told OR nondecept* OR “non decept*” OR nonconceal* OR “non conceal*” OR nonblind* OR “non blind*” OR “without deception” OR “without conceal*” OR “without blind*”) OR AB (told OR nondecept* OR “non decept*” OR nonconceal* OR “non conceal*” OR nonblind* OR “non blind*” OR “without deception” OR “without conceal*” OR “without blind*”) | 13263 |
| #5 | #3 AND #4 | 313 |
| #6 | TI ((open OR “open label”) N1 Placebo*) OR AB ((open OR “open label”) N1 Placebo*) | 74 |
| #7 | #5 OR #6 | 383 |
